# Historical Changes and Drivers of Aerosol Acidity in Switzerland under Emission Reduction and Its Implication of Regulation Policies

**DOI:** 10.1021/acs.est.5c15215

**Published:** 2026-07-13

**Authors:** Jun Zhang, Ali Waseem, Andrea Baccarini, Stylianos Kakavas, Christoph Hüglin, Athanasios Nenes

**Affiliations:** † Laboratory of Atmospheric Processes and Their Impacts, Ecole Polytechnique Federale de Lausanne, 1015 Lausanne, Switzerland; ‡ Institute of Chemical Engineering Sciences, Foundation for Research and Technology Hellas (FORTH/ICE-HT), 26504 Patras, Greece; § Empa, Swiss Federal Laboratories for Materials Science and Technology, 8600 Dübendorf, Switzerland; ∥ Laboratory of Atmospheric Processes and their Impacts, Institute of Chemical Engineering Sciences, Foundation for Research and Technology Hellas (FORTH/ICE-HT), 26504 Patras, Greece

**Keywords:** long-term aerosol pH, driving factor of aerosol acidity, reactive nitrogen, emission controls

## Abstract

Aerosol acidity is a key regulator of atmospheric processes, influencing particulate matter (PM) composition, toxicity, and the deposition of reactive nitrogen (Nr) to ecosystems. Emission controls in Europe have strongly reduced SO_*x*_ and NO_*x*_ but left NH_3_ largely unchanged, creating an imbalance between acidic and basic species that could shift aerosol pH and its impacts. Thermodynamic analysis of long-term observations (2008–2024) from Swiss monitoring sites combined with SHapley Additive exPlanations (SHAP) quantified aerosol pH and its key drivers. Annual mean pH shows a slight increasing trend, with consistently lower values in summer than in winter. SHAP analysis indicates that temperature drives seasonal pH variability at agricultural sites, whereas total ammonia (NH_3_^T^) dominates at the semialpine site. The Nr deposition analysis shows that the fast deposition of NH_3_^T^ predominates across both the lowland and Alpine regions, which increases local soil nitrogen burden. In contrast, PM has become increasingly insensitive to NH_3_ and more sensitive to HNO_3_, particularly in the agricultural sites. These results highlight that, although HNO_3_ precursor controls have effectively reduced PM pollution without the need for NH_3_ reductions, evermore significant ecological concerns remain from a lack of NH_3_ control. This underscores the need for coordinated reductions in both NO_*x*_ and NH_3_ emissions.

## Graphical abstract

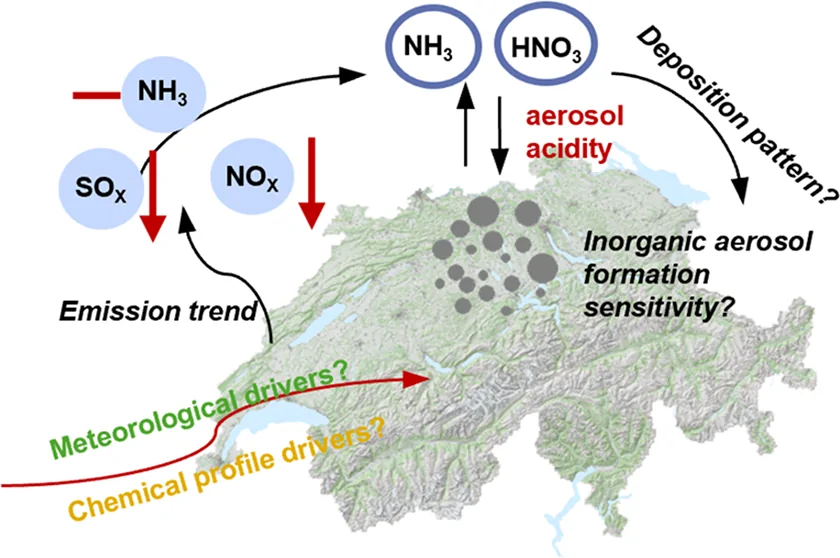

## Introduction

Atmospheric aerosols play an important role in air quality, climate, and human health.^1^ Aerosol acidity is a fundamental property that regulates numerous atmospheric processes, including the gas-particle partitioning of semivolatile species,^2^ the formation of secondary inorganic and organic aerosols,^3^ and the solubilization of trace metals.^4^ In the context of the nitrogen cycle, aerosol pH strongly affects the partitioning of reactive nitrogen (Nr) species such as ammonia (NH_3_), nitric acid (HNO_3_), ammonium (NH_4_^+^), and nitrate (NO_3_^–^), which differ markedly in their atmospheric lifetimes^5^ and deposition velocities.^3^ Excessive nitrogen can alter the plant growth rate,^6,7^ potentially diminishing carbon uptake and sequestration capacity,^8,9^ and also deteriorating groundwater quality.^10^ Determining the pH level and underlying drivers of aerosol acidity is therefore critical to understanding atmospheric processes and ecosystem impacts.

Aerosol composition such as sulfate (SO_4_^2–^), NO_3_^–^, NH_4_^+^, and nonvolatile cations (NVCs) has a fundamental impact on aerosol pH.^11–13^ Meanwhile, meteorological factors, such as temperature and relative humidity (RH), have been recognized as key factors in regulating the equilibrium and aerosol liquid water (ALW) concentration.^14–16^ These factors are inherently coupled, making it difficult to disentangle their relative contributions. For example, ALW is strongly influenced by hygroscopic components, yet the sensitivity of ALW and pH to composition depends critically on RH and temperature.^16,17^ More intrinsic coupling (such as meteorological conditions and partitioning of semivolatile components) has been demonstrated in various studies,^3,18,19^ underscoring the challenge of isolating the primary drivers of aerosol acidity.

Over the past few decades, emission control policies have substantially reduced SO_*x*_ and NO_*x*_ emissions across Europe, while NH_3_ emissions, largely from agricultural activities, have remained relatively unchanged. These emission reductions have altered particulate matter (PM) mass concentrations and chemical composition, but targeting only NO_*x*_ and SO_*x*_ and not NH_3_ raises important questions about how long-term emission trends translate into changes in pH, and how these, in turn, modulate PM sensitivity to precursor availability (sensitive to NH_3_, HNO_3_, both or none at all) and nitrogen dry deposition regime (fast/slow for NH_3_ and/or HNO_3_, respectively) across diverse environments. Therefore, long-term monitoring data on pH are critical for evaluating the effectiveness of emission control policies. Recent efforts have been undertaken in the United States,^19,20^ Canada,^21^ China,^17^ and Europe;^22,23^ yet significant gaps remain in the spatial and temporal coverage. In this study, we analyze existing long-term data from Swiss nitrogen deposition monitoring sites characteristic of the Swiss plateau, southern flanks, and semialpine regions to characterize the aerosol acidity levels, trends, and drivers, as well as the resulting PM sensitivity and nitrogen deposition regimes. This work provides a valuable data set in diverse observational sites under the effect of emission transition and evaluation of the effectiveness of emission control policies. The pH driver analysis offers a systematic and quantitative understanding of how the chemical profile and meteorology affect aerosol pH and further impact PM and Nr dry deposition.

## Methods

### Data Set Overview

The observational data set in this work were collected over 15 years from four sites in Switzerland at Payerne (PAY), Rigi-Seebodenalp (RIG), Magadino-Cadenazzo (MAG), and Beromünster (BRM). They are part of the National Air Pollution Monitoring Network (NABEL) in Switzerland. The measurements were conducted by the Federal Office for the Environment (FOEN) and Empa; information about NABEL and detailed station descriptions can be found at https://www.bafu.admin.ch/bafu/en/home/topics/air/luftbelastung/national-air-pollution-monitoring-network--nabel-.html. The PAY and BRM stations are situated in northern Switzerland at 489 and 797 m, respectively, surrounded by farming and agricultural lands, and represent rural background conditions in the Swiss plateau. The MAG station is representative of rural environments and is located at 203 m in the Magadino Plain of the Swiss southern flanks, surrounded by fields, with nearby industrial activity and a highway ∼2 km away. The RIG station is located at 1031 m in a pre-Alpine area of pastures and forest.

The data set used here are summarized in Table S1. Individual Nr species (NH_3_, NH_4_^+^, HNO_3_, NO_3_^–^) were collected using DELTA-MiniDenuder samplers at a 2-week resolution and analyzed with ion chromatography over 15 years at all sites. Daily resolution measurements of total nitrate NO_3_^T^ (sum of gas-phase HNO_3_ and particle-phase NO_3_^–^), total ammonia NH_3_^T^ (sum of gas-phase NH_3_ and particle-phase NH_4_^+^), and nonvolatile cations (NVCs, i.e., Ca^2+^, Na^+^, Mg^2+^, and K^+^) in total suspended particulate (TSP) were collected using impregnated filters and analyzed with ion chromatography at RIG and PAY. PM components NVCs, SO_4_^2–^, Cl^–^, and organic carbon in PM_10_ and PM_2.5_ were measured via filter sampling with varying resolutions and coverage, as shown in Table S1. Temperature, relative humidity, and ambient pressure are offered by MeteoSwiss, while the national emission data of NO*_x_*, SO_2_, and NH_3_ are from the European Monitoring and Evaluation Programme (EMEP) Centre on Emission Inventories and Projections (https://www.ceip.at/data-viewer-2/officially-reported-emissions-data). The uncertainties of the reported emissions are documented in each year’s Informative Inventory Report (IIR). For example, in the 2020 report, the overall uncertainties in activity data and emission factors for SO_2_, NO_*x*_, and NH_3_ are given as 8%, 13–14%, and 12%, respectively. The IIR can be downloaded at https://www.ceip.at/status-of-reporting-and-review-results

### Aerosol pH Estimation

Aerosol pH was estimated using the ISORROPIA-lite^24^ thermodynamic equilibrium model, a reduced-complexity version of the comprehensive ISORROPIA II model^25^ with extensions that include water uptake of hygroscopic organic species and their influence on inorganic partitioning. ISORROPIA-lite maintains consistency with ISORROPIA II but is optimized for computational efficiency by using precalculated binary activity coefficient tables. It also simplifies the assumed phase diagram of the aerosol, and assumes a metastable aerosol involving K^+^–Ca^2+^–Mg^2+^–NH_4_^+^–Na^+^–SO_4_^2–^–NO_3_^–^–Cl^–^–organics–H_2_O and simulates gas–aerosol equilibrium. A detailed description of ISORROPIA-lite can be found in Kakavas et al.^24^, and is avaliable at https://github.com/EPFL-LAPI/ISORROPIA-Iite.

ISORROPIA-lite requires input of ambient temperature, relative humidity, concentration of organic aerosol, NVCs, sulfate, total (aerosol and gas-phase) chloride (Cl^T^), ammonia (NH_3_^T^), and nitrate (NO_3_^T^). It calculates the partitioning of semivolatile inorganic species, liquid water content (LWC) associated with each species, and the concentration of hydrogen ions—from which pH is calculated (as the negative base 10 logarithm of the hydrogen ion concentration in the aerosol aqueous phase), reflecting the effective acidity of the particle-phase. The inferred pH values are realistic, as the thermodynamic model is able to reproduce the observed partitioning ratios of semivolatile species, which are very sensitive to acidity levels in the aerosol.^26^

The particles used as the primary input to ISORROPIA-lite here are in their majority of much smaller size than the 10 μm size cutoff associated with PM_10_ and thus largely in equilibrium with the gas phase. This is supported by observations of NO_3_^–^ and NH_4_^+^ in PM_2.5_, PM_10_, and TSP during periods of overlapping measurements at PAY. As shown in Figure S1, 73% of NO_3_^–^ and over 90% of NH_4_^+^ reside in PM_2.5_ compared to TSP. For PAY and RIG, daily NH_3_^T^ and NO_3_^T^ were used, while for MAG and BRM, biweekly measurements of the sum of individual species (NH_3_, NH_4_^+^, HNO_3_, NO_3_^–^) as NH_3_^T^ and NO_3_^T^ were used. Sulfate measurements were not available at MAG and BRM. For BRM, sulfate concentrations from PAY were used as a proxy, based on the similarity in station characteristics and tendency of sulfate to be spatially uniform owing to its regional character.^27^ For MAG, sulfate was estimated from measurements at Lugano (located ∼20 km south), applying a scaling factor derived from a linear regression of overlapping sulfate data collected over two years at both sites (Figure S2).

Organic mass (OM) was estimated from organic carbon (OC) using site-specific OM:OC ratios: 1.84 for RIG (based on measurements from a comparable Swiss mountain site^28^) and 1.95 for PAY.^29^ At MAG, OM was derived from two years of available organic measurements, with the average concentration applied to periods without observations. For BRM, the average OM concentration from PAY was used as a proxy because of the absence of measurement. A sensitivity analysis was conducted by varying OM by factors of 0.5 and 2, which showed a minimal impact on the calculated aerosol pH (Figure S3).

The NVCs in the sites considered, particularly Ca^2+^, Na^+^, and Mg^2+^, are strongly associated with agricultural, road, and mineral dust and are predominantly present in the coarse particle mode, while the other components, such as NO_3_^–^ and NH_4_^+^, as shown above, are mostly in the fine fraction (PM_2.5_). To determine the appropriate fraction of these cations that is consistent with the size range of other components for ISORROPIA-lite input, sensitivity tests were performed by varying their inclusion from 0% to 100% in 5% increments. Model outputs were evaluated based on the agreement between predicted and observed gas-particle partitioning of NH_3_/NH_4_^+^and HNO_3_/NO_3_^–^. Based on the minimum root-mean-square error (RMSE), optimal scaling factors of 0.2 and 0.4 were applied to Ca^2+^, Na^+^, and Mg^2+^ at PAY and RIG, respectively. No scaling factors are applied to K^+^ since its mass distribution is mainly within the PM_1_ range.^30,31^ Due to limited NVC data availability at BRM, the average NVC concentrations from PAY were used as a proxy. At MAG, only two years of NVC data are available; therefore, the missing NVC values were filled using the average from those 2 years at that site.

Prior to finalizing the data set used for analysis, data affected by dust events were excluded by removing the top 0.05% of PM_10_/PM_2.5_ ratios. Dust-rich particles are likely to fail the metastable and equilibrium assumptions required by ISORROPIA-lite for two reasons. First, high NVC levels in the aerosol may undergo crystallization under low RH and not satisfy the metastable aerosol assumption^25^ (although our data set does not have RH lower than 40% in the boundary layer, likely due to the daily/biweekly time resolution that averages out extremes). Second, coarse-mode dust enhances the partitioning of NO_3_^–^ into the coarse fraction, while NH_4_^+^ remains in the fine mode, creating two externally mixed aerosol populations that cannot be treated by simple equilibrium, as the coarse mode is generally not in equilibrium with the gas phase.^11^ Figure S4a shows the good linear relationship between simulated and observed partitioning of NH_4_^+^, with slopes close to 1, which suggests the reliability of the ISORROPIA-lite pH predictions for the data set used in this study. The correlation between simulated and measured NO_3_^–^ partitioning is weaker, a trend also reported in previous studies.^19,32^ This discrepancy likely arises from the low ambient HNO_3_ concentrations, which increase uncertainty, and from the inherent difficulty in accurately predicting activity coefficients. In addition, the averaged pH cannot capture short-term variations, such as diurnal pH changes driven by rapidly shifting meteorological conditions. These variations may have important implications for NVC chemistry, metal solubility, and related processes. Nevertheless, the averaged values reported here are representative of the broader seasonal and annual trends examined in this study and are appropriate for interpreting processes that act over coarser time scales, such as long-term emission reductions.

### Determining the Drivers of Aerosol pH

To quantify the contribution of each input variable to the calculated aerosol pH, SHAP (SHapley Additive exPlanations) analysis was applied directly to ISORROPIA-lite. SHAP explains model output by assigning an importance value to each feature based on its individual contribution to a model prediction. The SHAP value (Φ) for feature *i* is calculated as eq 1. It calculates the difference between the prediction with feature *i* included *f*_*S*∪{i}_(*x*_*S*∪{*i*}_) and that with the feature withheld *f*_*S*_(*x*_*S*_). Because the effect of withholding a feature depends on the context created by other features, the contribution of feature *i* is computed across all possible permutations of the feature set *S*⊆*F*\{*i*}.

1 $$\Phi_{i}=\Sigma_{S\subseteq F\backslash \left\{i\right\}}\frac{\left|S\right|!\left(\left|F\right|-\left|S\right|-1\right)!}{\left|F\right|!}\left[f_{S\cup \left\{i\right\}}\left(x_{S\cup \left\{i\right\}}\right)-f_{S}\left(x_{S}\right)\right]$$

The detailed information can be found elsewhere.^33^ In the context of aerosol pH estimation, SHAP estimates the marginal effect of each variable by evaluating the change in pH when that variable is added to all possible combinations of the other inputs using ISORROPIA-lite as the model engine. The resulting SHAP values represent the contribution of individual inputs to the predicted pH relative to the average conditions (unless specified otherwise).

Because these values are derived from ISORROPIA-lite evaluations, they reflect the physical and chemical relationships embedded in ISORROPIA-lite and provide a causally grounded and quantitatively consistent framework for assessing and comparing the dominant drivers of aerosol acidity across different sites and time periods. SHAP has been applied in atmospheric science to interpret the influence of complex drivers with models,^34–36^ and this study presents the first known application of SHAP to quantify the drivers of aerosol acidity.

## Results

### Temporal Changes of Emissions and Airborne Particles and Gas Concentrations

As shown in Figure 1, over the past decade in Switzerland, the SO_*x*_ and NO_*x*_ emissions have declined significantly by approximately 70% and 50%, respectively, while the NH_3_ emissions have decreased by only around 10%. These emission reductions directly impact corresponding air pollutant concentrations and aerosol levels. Over the past 15 years at our sites, airborne SO_4_^2–^ concentrations have dropped by 50% to 60%, falling below 1 μg m^–3^ in recent years. The NO_3_^T^, including both gas-phase HNO_3_ and particulate-phase NO_3_^–^, has reduced, in line with the NO_*x*_ emission reductions. NH_3_ concentrations in the air have remained relatively stable or even increased (Figure 1), while NH_4_^+^ concentrations dropped by over 50%. These shifts in NH_3_ and NH_4_^+^ cannot be explained by the observed change in emissions, but rather by changes in gas-particle partitioning of NH_3_^T^ owing to changes in temperature, humidity, and eventually other drivers of aerosol pH. The aspects most responsible for the observed partitioning are provided below.

**Figure 1:**
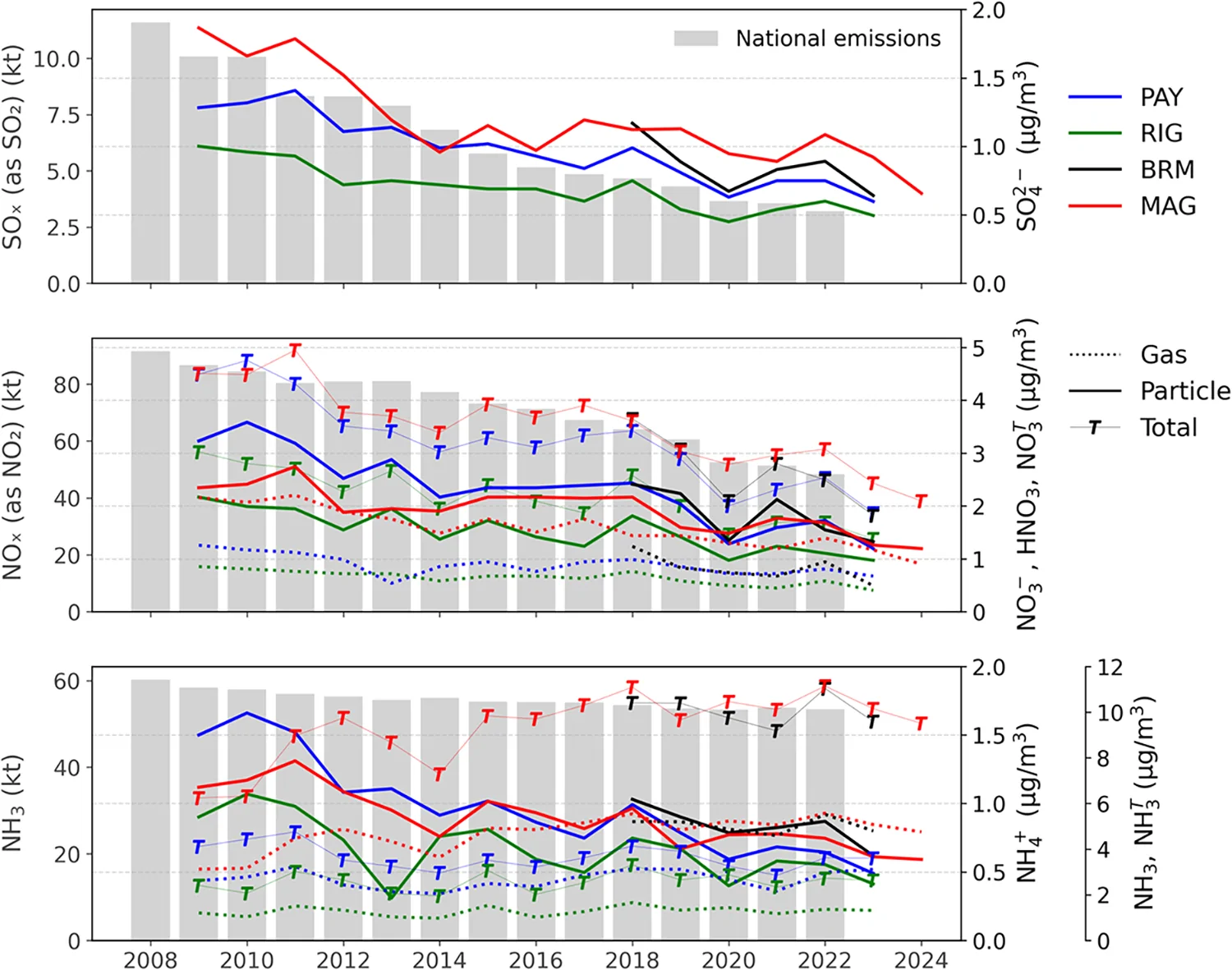
The time series of SO_4_^2–^, HNO_3_, NO_3_^–^, NH_3_, and NH_4_^+^ measured at four sites, compared with national emissions of SO*_x_*, NO*_x_*, and NH_3_. Solid lines represent particle-phase concentrations, while dashed lines indicate gas-phase concentrations. The letter *T* with lines denotes the total mass concentration of gas and particle phases. The national emissions are shown with gray bars.

### Yearly and Monthly Aerosol pH Variations

To investigate the aerosol pH response to the aerosol composition changes, aerosol pH at the four studied sites was estimated using ISORROPIA-lite. Among these sites, rural sites exhibited similar pH values, whereas the mountainous site (RIG) showed distinctly higher acidity (lower pH). The mean pH values inferred in order of decreasing pH are BRM (3.68 ± 0.43), followed by MAG (3.48 ± 0.51), PAY (3.34 ± 0.49), and RIG (2.99 ± 0.65). Figure 2a shows the aerosol pH yearly distribution over the past 15 years at each site. Statistically significant (*p* < 0.05) increasing trends were observed at RIG and MAG, with annual median pH increasing by 0.012 and 0.019 units per year, respectively. Although PAY and BRM also showed slight increases over time, trends did not reach statistical significance.

**Figure 2:**
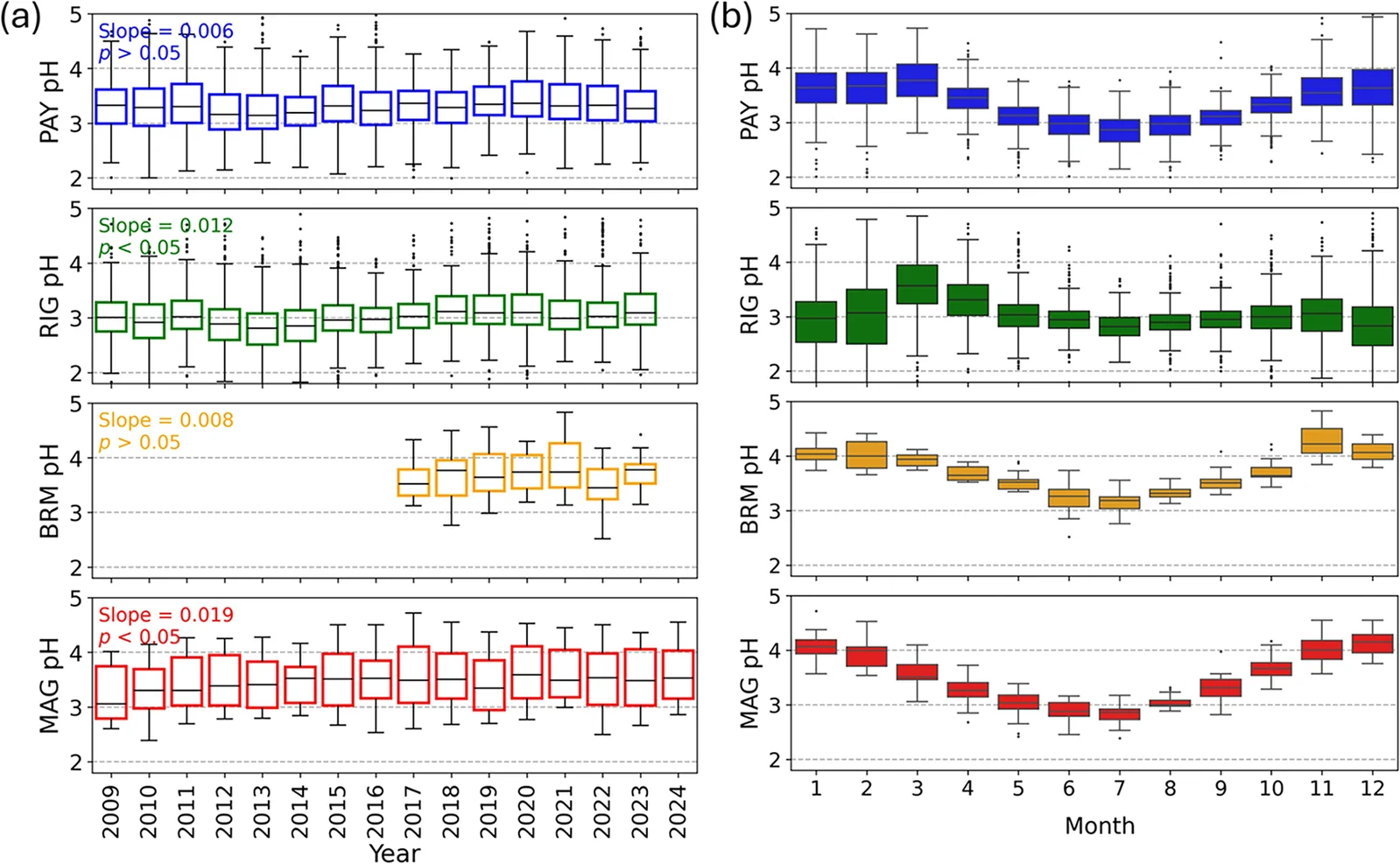
(a) Yearly and (b) monthly variations of aerosol pH at PAY, RIG, BRM, and MAG. The box represents the 25th and 75th percentiles and a horizontal bar denotes the median. In panel (a), the slopes indicate the linear fitting using yearly median values, with statistical significance denoted by the corresponding *p*-values. The *y*-axes were adjusted to enhance vertical resolution. The full set of data points is presented in Figure S5.

The pH range observed in this study is comparable to the pH reported at a long-term rural site in Germany.^22^ Despite substantial reductions in SO_4_^2–^ and NO_3_^–^ in Germany, aerosol pH has shown no decreasing trend over time.^22^ This is mainly attributed to the buffering effect of NH_3_. As SO_4_^2–^ and NO_3_^–^ decline, the reduced availability of acidic anions lowers the partitioning of NH_3_ into the particle. The same phenomenon has been observed for fine particle pH in the US,^20^ Canada,^15^ and the Po Valley in Italy.^23^ This shift in partitioning also explains why particulate NH_4_^+^ concentrations have declined more rapidly, while gas-phase NH_3_ levels have remained stable or slightly increased, despite 10% reductions in NH_3_ emissions.

Even though there are minor or no significant trends in annual pH, the seasonal and monthly variations are considerably stronger. In particular, aerosol pH is consistently lower during the warmest months of the year, as shown in Figure 2b. This pattern is consistent with observations from other regions and is thought to be driven by temperature variations.^15,21,37–39^ The monthly pH variations are approximately 2 units at BRM, MAG, and PAY, but only about 0.5 units at RIG. Notably, RIG exhibits several low-pH outliers (Figures 2 and S5), particularly during the cold season, likely influenced by intrusions of free-tropospheric air. To confirm this, the water vapor mixing ratio (*q*), which drops sharply in the free-tropospheric layer (FTL), was used as an indicator to determine the influence of FTL air masses. A threshold of *q* < 2.5 g kg^−1^ ^40^ was applied to indicate FTL using hourly metrological data at RIG. Since aerosol pH at RIG is available as daily averages, we calculated the daily fraction of time with *q* < 2.5 (*f*_*q*_) to represent the degree of FTL influence. Indeed, days with low pH (<2) coincide mostly with higher *f*_*q*_ values (FTL-dominated, points colored in red in Figure S6) and partially with lower *f*_*q*_ conditions (with some to significant planetary boundary influence, PBL, colored in blue). This shows that the low pH events can occur under both FTL and PBL conditions. A caveat is that under FTL influence, hourly RH occasionally drops below 40% despite higher daily averages, which may increase the uncertainty of the reported pH values. Further, we compared input variables for days with pH < 2 versus days with pH between 2 and 4, stratified by FTL (*f*_*q*_ > 0.6) and PBL (*f*_*q*_ ≤ 0.6) conditions (Figure S7). Under FTL influence, key acidic components, SO_4_^2–^ and NO_3_^T^ concentrations are significantly elevated by long-range transport, which may tend to lower the aerosol pH owing to a lack of NH_4_^+^ for the fine mode aerosol and insufficient NVCs to partially neutralize sulfate. Conversely, when the boundary layer arises, the low-pH outliers are primarily associated with substantially reduced NH_3_^T^ concentrations in winter, likely owing to the expected low NH_3_ emission from reduced anthropogenic and biological activities. The reduced NH_3_^T^ in turn is less effective in neutralizing sulfates, leading to a decrease in pH.

### What Drives Aerosol pH Variations?

The above analysis points to factors that can influence aerosol pH, but no conclusive statements can be made on which factors really matter and by how much. While the effects of individual input variables on aerosol pH can be separately quantified, some variables are correlated such as temperature and RH, so that their impact on aerosol pH is often a result of their combined effects and challenging to unravel. Previous efforts to identify pH drivers have often relied on prior knowledge of dominant acid–base buffering pairs,^15,18,41^ which may neglect the influence of other important species, or on extensive sensitivity testing,^32,38,39,42^ which can be computationally demanding, assume linearity or independence of input variables. To systematically investigate aerosol pH variations across sites and time periods, we coupled ISORROPIA-Lite with SHAP analysis to quantify the marginal effect of individual input variables on pH, relative to the average condition (or any other reference condition). This approach is broadly applicable without constraints imposed by specific buffering regimes, capable of capturing causal relationships, and powerful for deconvolving feature contributions even when variables are interdependent, such as temperature and RH.

Figure 3 presents the decomposition of monthly aerosol pH variability into meteorological drivers and chemical constituents. The aerosol pH monthly variability at three rural sites, PAY, MAG, and BRM, has similar driving factors, which are in order of importance: temperature, followed by NH_3_^T^ (Figure S8 for MAG and BRM). These two factors were also identified as the most important drivers at a rural site in Germany using an independent evaluation method.^22^ Elevated temperatures during warmer months shift the thermodynamic equilibrium of semivolatile species such as NH_3_/NH_4_^+^ and HNO_3_/NO_3_^–^ toward the gas phase.^25^ As NH_3_ tends to be more volatile than HNO_3_, the loss of particle-phase NH_4_^+^ exceeds that of NO_3_^–^, resulting in a net decrease in aerosol pH as sulfate (and some nitrate) remains and dissociates in aerosol water to form H^+^. For similar reasons, NH_3_^T^ is identified as the second most important driver at those sites: NH_3_^T^, when it partitions to the aerosol phase, reacts with free protons in the aerosol aqueous phase, forming aerosol NH_4_^+^ and shifting the water dissociation equilibrium toward higher pH. When NH_3_^T^ level decreases, its diminished buffering capacity results in a negative SHAP contribution to pH. SO_4_^2–^ was the third key variable at PAY and MAG, linked to the ionic species with very low volatility and capacity for strong acidity. As SHAP values indicate how each variable shifts the estimated pH relative to the mean estimation over the analyzed period, higher SO_4_^2–^ generally lowers aerosol pH and shows negative SHAP values. In recent years, the pronounced decline in SO_4_^2–^ concentrations have produced more frequent positive SHAP values, reflecting a relative pH increase due to a marked reduction in SO_4_^2–^concentration. For example, at PAY, around 40% of daily SO_4_^2–^concentrations since 2020 have been below 0.5 μg m^–3^. This has led the available SO_4_^2–^ to be more neutralized by NH_4_^+^ and NVCs, causing low-sulfate conditions to have elevated pH compared to high-sulfate conditions. Additionally, the reduction of SO_4_^2–^ could lead to the increased partitioning of NO_3_^–^ (in the form of ammonium nitrate) in its place, which is less acidic and whose associated water content further dilutes H^+^ and increases pH. Although the simultaneous reduction of NO_3_^–^ concentration means the absolute water content from NH_4_NO_3_ did not show an increase (Figure S10), the relative contribution of nitrate water over water from sulfates increases. This means nitrate retains more water per unit aerosol than sulfate would. This shift toward a greater fraction of nitrate-associated water dilutes the proton concentration in the liquid phase and consequently increases the pH.

**Figure 3:**
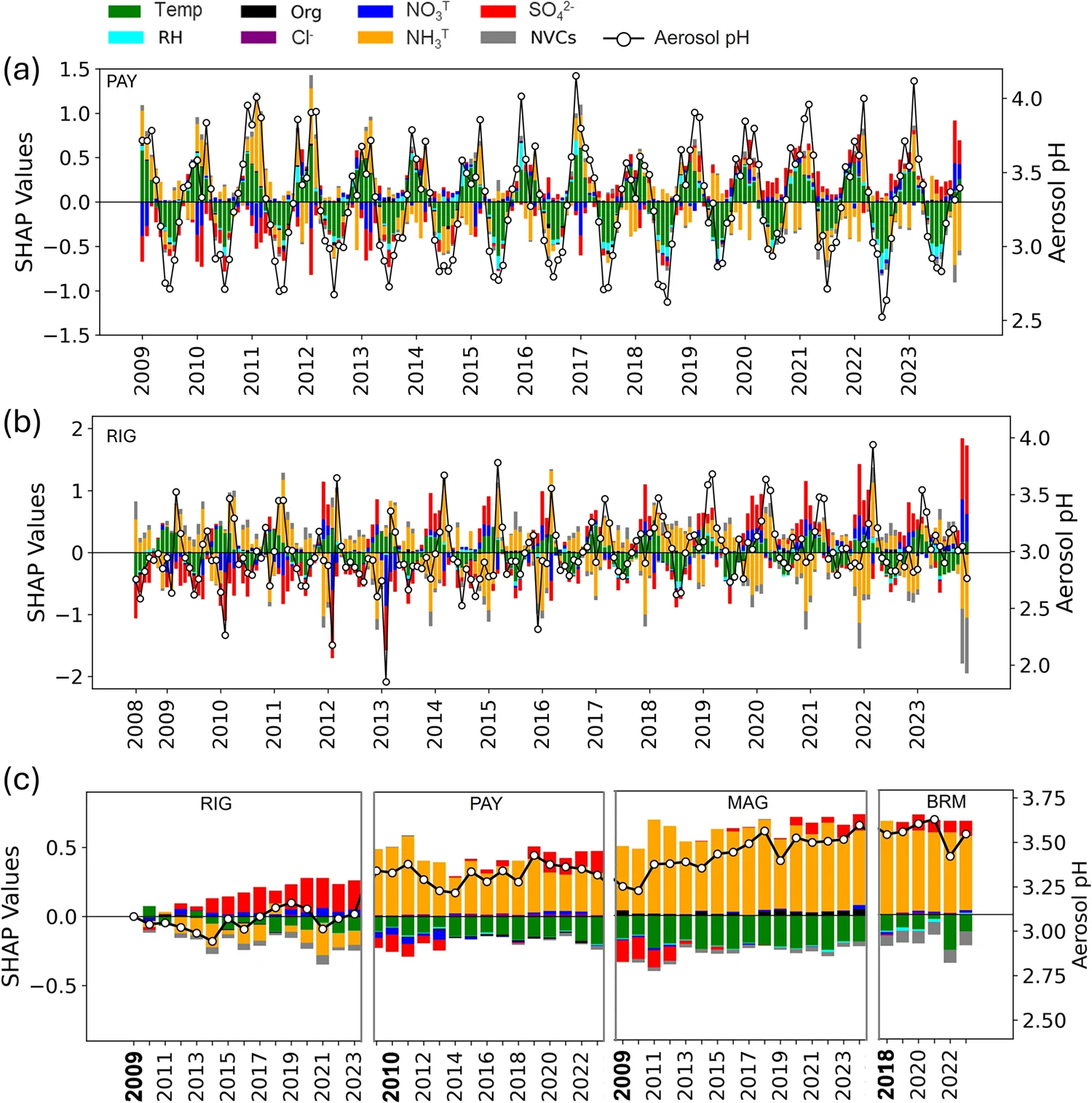
Monthly and yearly SHAP-derived contributions of key variables to aerosol pH. The SHAP values of temperature (Temp), relative humidity (RH), total nitrate (NO_3_^T^), total ammonia (NH_3_^T^), sulfate (SO_4_^2–^), chloride (Cl^–^), organics (Org), and nonvolatile cations (NVCs) are shown as stacked bars. Monthly contributions of variables to the average condition at PAY and RIG are shown at (a) PAY and (b) RIG, respectively. The comparison of four sites on a yearly basis is shown in panel (c) using the condition of RIG in 2009 as the base condition.

The mountain site RIG exhibited distinct drivers of aerosol pH compared to the drivers for the low-altitude rural sites. At RIG, NH_3_^T^ was the dominant contributor to pH variability. This is attributed to a more pronounced seasonal cycle in NH_3_^T^ compared to other sites, with low concentrations in winter and peak levels in summer (Figure S9). Temperature ranked as the second most important factor. Although the average temperature at RIG is approximately 2 °C lower than at PAY, its seasonal variation amplitude is similar. However, the elevated NH_3_^T^ in summer at RIG offsets the effect of temperature in decreasing the pH, resulting in a weaker seasonal pattern in pH at this site (Figure 2). SO_4_^2–^ as the third key factor, behaved similarly to that at the rural sites. Its decreasing concentrations in recent years, relative to other precursors, have diminished its role in acidifying aerosols. NO_3_^T^ consistently exhibited low SHAP values across all sites, indicating minimal influence on aerosol pH variability. This is expected in NH_3_-rich environments, where HNO_3_ is efficiently neutralized by excess NH_3_ to form NH_4_NO_3_, contributing little to free proton concentrations. This is expected in NH_3_-rich environments, where HNO_3_ is efficiently neutralized by excess NH_3_ to form NH_4_NO_3_, contributing little to free proton concentrations. In addition, the amount of nitrate-associated water remains relatively stable over the studied years (Figure S10), providing an unchanged dilution effect via water uptake. Together, these two factors suggest that NO_3_^T^ has only a weak effect on pH, reflected by its low SHAP values.

The SHAP analysis allows direct quantification of the relative contributions of individual input variables to the aerosol pH, facilitating cross-site and temporal comparisons. As shown in Figure 3c, to compare the pH at four sites, we chose the meteorological conditions and chemical composition at RIG in 2009 as the common baseline for comparison. Spatially, higher pH values observed at the agricultural sites (PAY, MAG, and BRM) relative to RIG are primarily explained by the higher NH_3_^T^ levels, which offset the pH-lowering effect of higher temperatures at lower altitudes. Temporally, SO_4_^2–^ initially contributed to a lower pH. However, as its concentration declined over the past decade, its influence shifted toward supporting pH increases compared to the earlier high-SO_4_^2–^ conditions. This has also been observed in eastern China, where a reduction in SO_4_^2–^ led to a 0.38 unit increase in pH over 10 years.^43^

### Contribution of HNO_3_ and NH_3_ to PM Sensitivity and Deposition Regimes

The sensitivities of PM mass to HNO_3_ and NH_3_ are determined by the thermodynamic conditions of the aerosol system. To assess this sensitivity, we applied the framework developed by Nenes et al.,^3^ which classifies aerosol response into four regimes based on aerosol pH and liquid water content at given temperatures, i.e., HNO_3_-sensitive only, NH_3_-sensitive only, HNO_3_- and NH_3_-sensitive, and insensitive to both. Following Nenes et al.,^3^ a regime is considered sensitive when more than 10% of the precursor (NO_3_^T^ or NH_3_^T^) can partition into the particle phase.

As shown in Figure 4, PM sensitivity regimes were characterized across space and time in Switzerland. Overall, the dominant PM sensitivity regimes are HNO_3_-sensitive (blue bars with dashed lines), both HNO_3_- and NH_3_-sensitive (purple bars with dashed lines). However, long-term trends at PAY, MAG, and RIG indicate a transition: PM is becoming increasingly sensitive to HNO_3_, while the contribution of both HNO_3_ and NH_3_ sensitivity is decreasing. This trend is particularly pronounced at MAG, where in recent years, PM has become predominantly HNO_3_-sensitive. This shift can be attributed to the high aerosol pH and low liquid water content, driven by declining NO_3_^–^ and SO_4_^2–^ concentrations, which in turn promote NH_3_ remaining in the gas phase. These findings suggest that a reduction in HNO_3_ precursors would be particularly effective in lowering PM concentrations. Due to the nonlinear relationship between nitrate and NO_X_, further analysis is needed to determine appropriate reduction measures. For example, at agricultural sites with high NH_3_ levels, HNO_3_ formation could be sensitive to NO_*x*_ emission or oxidant levels, which could be limited by volatile organic compound (VOC) emissions. In the absence of VOC measurement, high NO_2_ could be used as an indicator of high VOC conditions.^44^ As shown in Figure S11, nitrate formation at PAY tends to be VOC-sensitive in cold seasons due to oxidant competition, whereas in warm seasons it is more likely to be NO_*x*_-sensitive, following the scheme proposed by Dang et al.^44^

**Figure 4:**
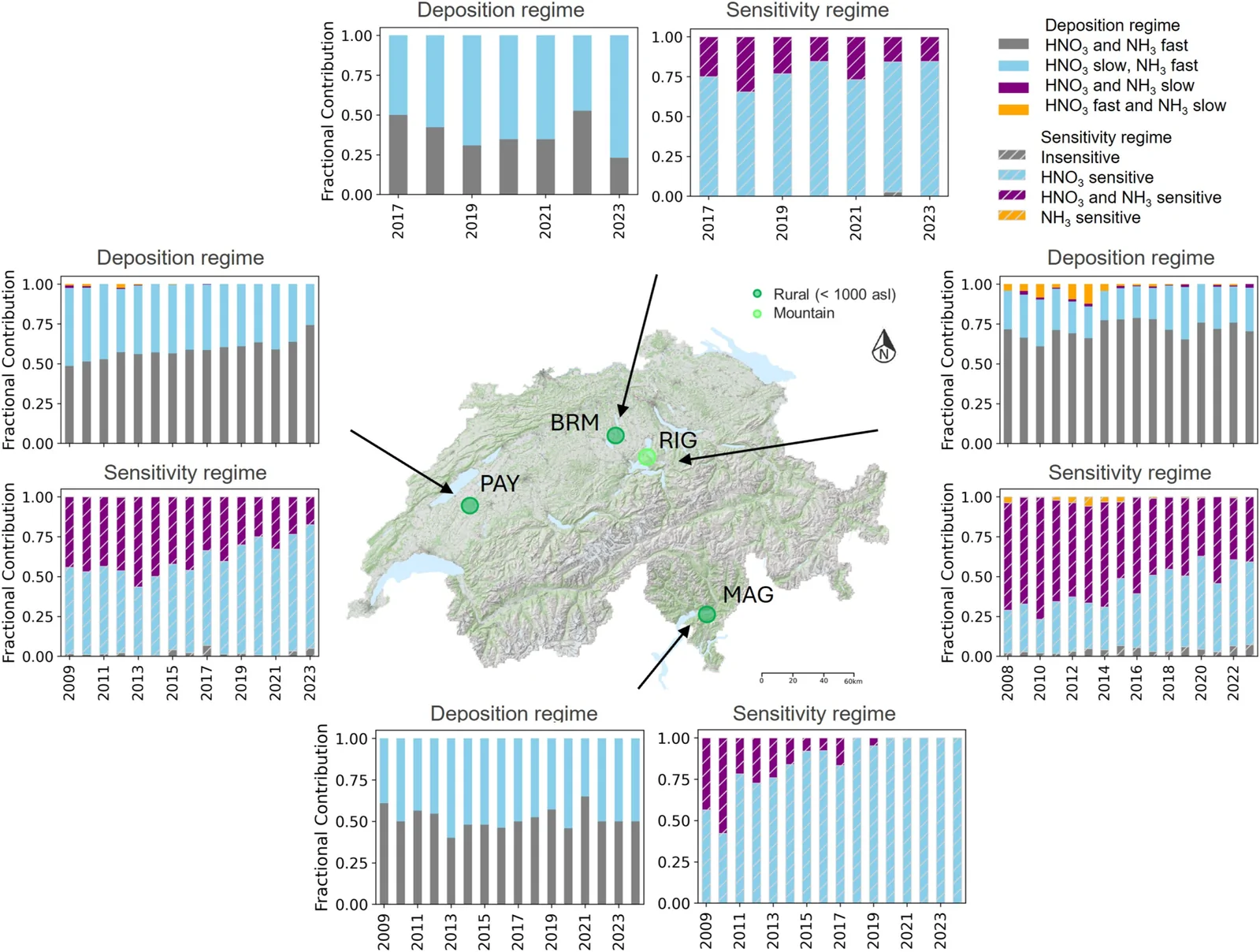
The yearly deposition regimes and sensitivity regimes at four sites in Switzerland. The map shows the locations of the four monitoring sites in Switzerland, indicated by green circles. Dark green and light green denote rural and mountainous sites, respectively. The yearly distribution of deposition and sensitivity regimes at each site is shown, with regime fractions color-coded by types.

To characterize the dry deposition regime of the aerosol owing to changes in aerosol pH and liquid water content, we use the framework of Nenes et al.;^5^ if more than 90% of a species (HNO_3_ or NH_3_) is in the gas phase, it is considered to undergo “fast deposition”. In the opposite case, slow deposition is also associated with a tendency to accumulate nitrogen species in the aerosol phase and the boundary layer (potentially leading to haze events). Therefore, four regimes of dry deposition velocity are (1) HNO_3_ and NH_3_ fast, (2) HNO_3_ slow and NH_3_ fast, (3) HNO_3_ slow and NH_3_ slow, and (4) HNO_3_ fast and NH_3_ slow. As shown in Figure 4, the deposition of NH_3_ is predominantly “fast”, meaning that NH_3_ emissions redeposit relatively closely to their source. It also means that NH_4_^+^ tends to accumulate less in the atmosphere unless the aerosols are extremely acidic. The deposition of HNO_3_ transits between slow and fast, driven by seasonal variation, with warm-season deposition being fast due to the reduced pH. Wintertime slow nitrate deposition rates also imply that nitrate can accumulate in the boundary layer, contributing to regional haze and depositing far away from where it is produced.

Given the decreasing emission trends of NO_*x*_ and NH_3_ in Figure 1, a reduction in deposition of the corresponding species is expected. Previous studies have documented decreasing trends in oxidized nitrogen deposition in Switzerland from 2000 to 2017 (−1.6% to −1.8% per year), which responds less to the NO_*x*_ emission decreases (−2.4% per year).^45^ The rising share of fast-depositing HNO_3_ from our deposition regime analysis suggests that NO_3_^T^ shifts more to the gas phase and accumulates less in the atmosphere. This could partially offset the expected linear decrease of nitrogen deposition due to the reductions in NO_*x*_ emissions. On the other hand, reduced nitrogen deposition (dominated by NH_3_) has not shown a consistent decline and has even increased at MAG, under the condition of −0.4% per year reduction in NH_3_ emission. This could be explained by the fast deposition of NH_3_, which is likely sufficient to counterbalance minor emission reductions, potentially leading to stagnation or even increases in NH_3_ deposition. This underscores the need for more stringent NH_3_ control measures to effectively manage nitrogen deposition impacts on the ecosystem health.

### Implications

Our analysis of aerosol pH, liquid water content, and the resulting PM sensitivity regimes suggests that continued reductions in HNO_3_ through NO_*x*_ or VOC emission control remain an effective strategy for mitigating PM levels in Switzerland, as PM formation is currently most sensitive to HNO_3_ availability—as nitrate is the “limiting factor” for aerosol formation. This approach has already been implemented through national air quality policies, with substantial declines in NO_*x*_ and SO_*x*_ emissions over the past decades (Figure 1). As a result, both particulate NO_3_^–^ and NH_4_^+^ concentrations have substantially declined, in proportion almost to the NO_*x*_ emission reduction, the latter due to reduced availability of acidic anions for neutralization, despite only modest reductions in NH_3_ emissions.

Our results show that NH_3_ deposition remains predominantly fast in both the Swiss plateau, southern flanks, and the pre-Alpine region, posing significant risks to sensitive ecosystems where critical nitrogen loads have already been exceeded.^45^ By contrast, HNO_3_ shows a larger fraction of slow deposition in the plateau and southern flanks, suggesting that its impacts extend beyond local ecosystems to a regional scale, whereas in the pre-Alpine region, both NH_3_ and HNO_3_ from local sources deposit fast near sources. These distinct deposition regimes highlight that reducing NH_3_ emissions will be especially effective in lowering nitrogen loads in nearby ecosystems, particularly in the lowland plains, while reducing HNO_3_ precursors, apart from impacts on air quality, would benefit ecosystems downwind.

Under future climate warming, aerosol pH in Switzerland is expected to moderate the previously observed increasing trend, as higher temperatures promote the partitioning of semivolatile species into the gas phase. Moreover, continental RH is projected to decrease under climate change,^46–48^ leading to lower aerosol liquid water, which may further mitigate the upward trend in pH over the past decade. This will likely decrease overall PM mass but enhance the deposition rate of reactive nitrogen. For instance, HNO_3_ deposition is expected to shift toward a larger fraction of fast deposition, a trend already observed at PAY, thereby reducing regional export while increasing the local oxidized nitrogen burden. More importantly, NH_3_ deposition will remain the largest contributor to dry deposition due to its largely uncontrolled emissions, further intensifying nitrogen loads in sensitive ecosystems. These findings highlight that considering climate–chemistry interactions are beneficial when designing future emission control strategies. In particular, although the lack of NH_3_ emission control had a relatively modest impact on PM levels, its rapid deposition means that reducing NH_3_ emissions is crucial for mitigating the impacts of excess nitrogen burden on ecosystems, groundwater, and the release of the greenhouse N_2_O.

## Supplementary Material



## Data Availability

Script and example of input of SHAP analysis are available. ISORROPIA-lite is avaliable at https://github.com/EPFL-LAPI/ISORROPIA-Iite.

## Notes

Detailed list of sampling methods and the corresponding time-resolved data availability; scatter plots for ISORROPIA-lite output verification; analysis of planetary boundary layer influences; SHAP-derived results for MAG and BRM; time series of NH_3_^T^ at the sampling sites (PDF)

## References

[1] Pöschl U. (2005). Atmospheric
Aerosols: Composition, Transformation, Climate and Health Effects. Angew. Chem., Int. Ed..

[2] Wallace B. J., Mongeau M. L., Zuend A., Preston T. C. (2023). Impact of pH on
Gas-Particle Partitioning of Semi-Volatile Organics in Multicomponent
Aerosol. Environ. Sci. Technol..

[3] Nenes A., Pandis S. N., Weber R. J., Russell A. (2020). Aerosol pH and liquid
water content determine when particulate matter is sensitive to ammonia
and nitrate availability. Atmos. Chem. Phys..

[4] Fang T., Guo H., Zeng L. (2017). Highly Acidic Ambient Particles, Soluble Metals,
and Oxidative Potential: A Link between Sulfate and Aerosol Toxicity. Environ. Sci. Technol..

[5] Nenes A., Pandis S. N., Kanakidou M. (2021). Aerosol acidity and
liquid water content regulate the dry deposition of inorganic reactive
nitrogen. Atmos. Chem. Phys..

[6] Vallicrosa H., Johnson K. M., Gessler A. (2024). Temperature and leaf
form drive contrasting sensitivity to nitrogen deposition across European
forests. Sci. Total Environ..

[7] Braun S., Schindler C., Rihm B. (2017). Growth trends of beech and Norway
spruce in Switzerland: The role of nitrogen deposition, ozone, mineral
nutrition and climate. Sci. Total Environ..

[8] Liu M., Shang F., Lu X. (2022). Unexpected response
of nitrogen deposition to nitrogen oxide controls and implications
for land carbon sink. Nat. Commun..

[9] Flechard C. R., Ibrom A., Skiba U. M. (2020). Carbon–nitrogen
interactions in European forests and semi-natural vegetation –
Part 1: Fluxes and budgets of carbon, nitrogen and greenhouse gases
from ecosystem monitoring and modelling. Biogeosciences.

[10] Covatti G., Li K.-Y., Podgorski J., Winkel L. H. E., Berg M. (2025). Nitrate contamination
in groundwater across Switzerland: Spatial prediction and data-driven
assessment of anthropogenic and environmental drivers. Sci. Total Environ..

[11] Guo H., Nenes A., Weber R. J. (2018). The underappreciated
role of nonvolatile
cations in aerosol ammonium-sulfate molar ratios. Atmos. Chem. Phys..

[12] Shi G., Xu J., Peng X. (2017). pH of Aerosols in a
Polluted Atmosphere: Source
Contributions to Highly Acidic Aerosol. Environ.
Sci. Technol..

[13] Pye H. O. T., Nenes A., Alexander B. (2020). The acidity of atmospheric
particles and clouds. Atmos. Chem. Phys..

[14] Guo H., Xu L., Bougiatioti A. (2015). Fine-particle water and pH in the southeastern
United States. Atmos. Chem. Phys..

[15] Tao Y., Murphy J. G. (2021). Simple Framework
to Quantify the Contributions from
Different Factors Influencing Aerosol pH Based on NHx Phase-Partitioning
Equilibrium. Environ. Sci. Technol..

[16] Zheng G., Su H., Wang S. (2020). Multiphase buffer theory explains contrasts
in atmospheric aerosol acidity. Science.

[17] Nah T., Lam Y. H., Yang J., Yang L. (2023). Long-term trends and
sensitivities of PM2.5 pH and aerosol liquid water to chemical composition
changes and meteorological parameters in Hong Kong, South China: Insights
from 10-year records from three urban sites. Atmos. Environ..

[18] Duan, X. ; Zheng, G. ; Chen, C. ; Zhang, Q. ; He, K. Driving factors of aerosol acidity: a new hierarchical quantitative analysis framework and its application in Changzhou, China. 2024 10.5194/egusphere-2024-3584.

[19] Guo H., Sullivan A. P., Campuzano-Jost P. (2016). Fine particle pH and
the partitioning of nitric acid during winter in the northeastern
United States. J. Geophys. Res. Atmos..

[20] Weber R. J., Guo H., Russell A. G., Nenes A. (2016). High aerosol
acidity despite declining
atmospheric sulfate concentrations over the past 15 years. Nat. Geosci..

[21] Tao Y., Murphy J. G. (2019). The sensitivity of PM_2.5_ acidity to meteorological
parameters and chemical composition changes: 10-year records from
six Canadian monitoring sites. Atmos. Chem.
Phys..

[22] Pratap V., Hennigan C. J., Stieger B. (2025). Climatology of aerosol
pH and its controlling factors at the Melpitz continental background
site in Central Europe. Atmos. Chem. Phys..

[23] Paglione M., Decesari S., Rinaldi M. (2021). Historical Changes in
Seasonal Aerosol Acidity in the Po Valley (Italy) as Inferred from
Fog Water and Aerosol Measurements. Environ.
Sci. Technol..

[24] Kakavas S., Pandis S. N., Nenes A. (2022). ISORROPIA-Lite: A Comprehensive Atmospheric
Aerosol Thermodynamics Module for Earth System Models. Tellus B.

[25] Fountoukis C., Nenes A. (2007). ISORROPIA II: a computationally
efficient thermodynamic equilibrium
model for K+–Ca2+–Mg2+–NH4+–Na+–SO42––NO3––Cl––H2O
aerosols. Atmos Chem. Phys..

[26] Guo H., Xu L., Bougiatioti A. (2015). Fine-Particle water and pH in the southeastern
United States. Atmos. Chem. Phys..

[27] Malm W. C., Schichtel B. A., Pitchford M. L., Ashbaugh L. L., Eldred R. A. (2004). Spatial
and monthly trends in speciated fine particle concentration in the
United States. J. Geophys. Res. Atmos..

[28] Cozic J., Verheggen B., Weingartner E. (2008). Chemical composition
of free tropospheric aerosol for PM1 and coarse mode at the high alpine
site Jungfraujoch. Atmos. Chem. Phys..

[29] Bozzetti C., Daellenbach K. R., Hueglin C. (2016). Size-Resolved Identification,
Characterization, and Quantification of Primary Biological Organic
Aerosol at a European Rural Site. Environ. Sci.
Technol..

[30] Rogula-Kozłowska W. (2016). Size-segregated
urban particulate matter: mass closure, chemical composition, and
primary and secondary matter content. Air Qual.,
Atmos. Health.

[31] Kozáková J., Pokorná P., Vodička P. (2019). The influence of local
emissions and regional air pollution transport on a European air pollution
hot spot. Environ. Sci. Pollut. Res..

[32] Ding J., Zhao P., Su J. (2019). Aerosol pH and its driving
factors in Beijing. Atmos. Chem. Phys..

[33] Lundberg, S. M. ; Lee, S.-I. A Unified Approach to Interpreting Model Predictions Curran Associates Inc.: Long Beach, California, USA pp 4768–4777.

[34] Peng X., Xie T. T., Tang M. X. (2023). Critical Role of Secondary
Organic Aerosol in Urban Atmospheric Visibility Improvement Identified
by Machine Learning. Environ. Sci. Technol.
Lett..

[35] Zhang Y., Liu Y., Ma W. (2025). Changing aerosol chemistry is redefining HONO
sources. Nat. Commun..

[36] Hu Q., Moon J., Kim H. (2024). Understanding the Driving Forces
of Summer PM1 Composition in Seoul, Korea, with Explainable Machine
Learning. ACS EST Air.

[37] Arangio A. M., Shahpoury P., Dabek-Zlotorzynska E., Nenes A. (2022). Seasonal Aerosol Acidity,
Liquid Water Content and Their Impact on Fine Urban Aerosol in SE
Canada. Atmosphere.

[38] Jia S., Chen W., Zhang Q. (2020). A quantitative analysis
of the driving factors affecting seasonal variation of aerosol pH
in Guangzhou, China. Sci. Total Environ..

[39] Sharma B., Jia S., Polana A. J. (2022). Seasonal variations in aerosol acidity
and its driving factors in the eastern Indo-Gangetic Plain: A quantitative
analysis. Chemosphere.

[40] Foskinis R., Gao K., Gini M. I. (2024). The Influence of the Planetary Boundary
Layer on the Atmospheric State at an Orographic Site at the Eastern
Mediterranean. Tellus B.

[41] Wang G., Tao Y., Chen J. (2022). Quantitative Decomposition of Influencing Factors
to Aerosol pH Variation over the Coasts of the South China Sea, East
China Sea, and Bohai Sea. Environ. Sci. Technol.
Lett..

[42] Liu P., Zhao X., Zhang C. (2021). Fine particle pH and
its influencing factors during summer at Mt. Tai: Comparison between
mountain and urban sites. Atmos. Environ..

[43] Zhou M., Zheng G., Wang H. (2022). Long-term trends and
drivers of aerosol pH in eastern China. Atmos.
Chem. Phys..

[44] Dang R., Jacob D. J., Zhai S. (2023). Diagnosing the Sensitivity
of Particulate Nitrate to Precursor Emissions Using Satellite Observations
of Ammonia and Nitrogen Dioxide. Geophys. Res.
Lett..

[45] Kosonen Z., Schnyder E., Hiltbrunner E. (2019). Current atmospheric
nitrogen deposition still exceeds critical loads for sensitive, semi-natural
ecosystems in Switzerland. Atmos. Environ..

[46] Allan R. P., Douville H. (2024). An even drier future
for the arid lands. Proc. Natl. Acad. Sci. U.S.A..

[47] Douville H., Willett K. M. (2023). A drier than expected future, supported
by near-surface
relative humidity observations. Sci. Adv..

[48] MeteoSwiss & ETH Zurich . Climate CH2025 - Scientific Report Federal Office of Meteorology and Climatology MeteoSwiss: Zurich; 2025.

