# Supplementary material for: Historical Changes and Drivers of Aerosol Acidity in Switzerland under Emission Reduction and Its Implication of Regulation Policies
Source: Environ Sci Technol. 2026 Jul 13;60(40):28671–80. doi: 10.1021/acs.est.5c15215 (PMC13674077; doi:10.1021/acs.est.5c15215)
Supplement: Supplementary file 1 [file es5c15215_si_001.pdf]

Supporting information

Historical Changes and Drivers of Aerosol Acidity in Switzerland under emission reduction and its implication of regulation policies

Jun Zhang [a*], Ali Waseem [a], Andrea Baccarini [a], Stylianos Kakavas [b], Christoph Hüglin [c], and Athanasios Nenes [a,d*]

[a] Laboratory of Atmospheric Processes and their Impacts, Ecole Polytechnique Federale de Lausanne, 1015 Lausanne, Switzerland

[b] Institute of Chemical Engineering Sciences, Foundation for Research and Technology Hellas (FORTH/ICE-HT), 26504 Patras, Greece

[c] Empa, Swiss Federal Laboratories for Materials Science and Technology, 8600 Dübendorf, Switzerland

[d] Laboratory of Atmospheric Processes and their Impacts, Institute of Chemical Engineering Sciences, Foundation for Research and Technology Hellas (FORTH/ICE-HT), 26504 Patras, Greece

[*] Email: jun.zhang@epfl.ch

[*] Email: athanasios.nenes@epfl.ch

Table S1. Overview chemical species availability and sampling methods at sampling stations.

Parameter	Rigi-Seebodenalp (RIG)	Payerne (PAY)	Magadino-Cadenazzo (MAG)	Beromünster (BRM)
**Station type**	Mountain	Rural	Rural	Rural
**Elevation (m)**	1031	489	203	797
**Study period**	2008 - 2023	2009 - 2023	2009 - 2024	2017 - 2023
**Measurement Method**				
NH_3, NH_4^+, HNO_3, NO_3^- (aerosol phase in TSP)	DELTA-MiniDenuder (2-week)	DELTA-MiniDenuder (2-week)	DELTA-MiniDenuder (2-week)	DELTA-MiniDenuder (2-week)
$\sum(HNO_3 + NO_3^-)$ (aerosol phase in TSP)	Impregnated filters (daily)	Impregnated filters (daily)	n.a.	n.a.
$\sum(NH_3 + NH_4^+)$ (aerosol phase in TSP)	Impregnated filters (daily)	Impregnated filters (daily)	n.a.	n.a.
NVCs in TSP	Impregnated filters (daily)	Impregnated filters (daily)	n.a.	n.a.
NVCs in PM_{10}	n.a.	Filter (4-day in 2008–2009, 2018–2019)	Filter (4-day in 2008–2009, 2018–2019)	Proxy (PAY mean)
SO_4^{2-}, Cl^- in PM_{10}	Filter (daily)	Filter (daily)	Proxy (LUG, 1-month)	Proxy (PAY, 2-week)
SO_4^{2-}, Cl^-, NH_4^+, NO_3^- in PM_{10} and $PM_{2.5}$	n.a.	Filter (4-day in 2008–2009, 2018–2019)	Proxy (LUG, 1-month)	n.a.
Organic carbon in $PM_{2.5}$	Filter (daily)	Filter (daily)	Filter (4-day in 2008–2009, 2018–2019)	n.a.
Organic carbon in PM_{10}	n.a.	Filter (4-day in 2008–2009, 2018–2019)	Filter (4-day in 2008–2009, 2018–2019)	Proxy (PAY mean)

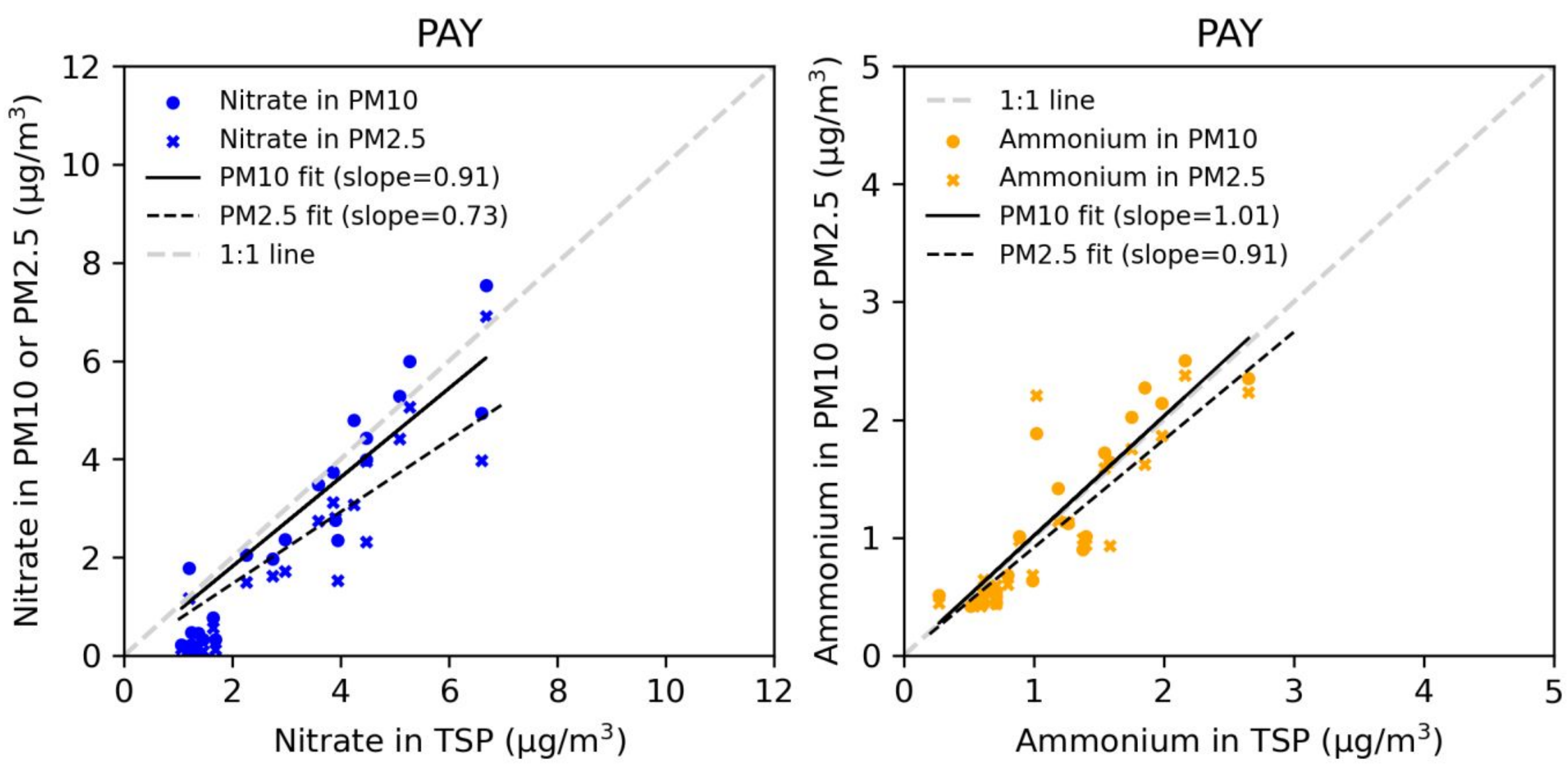

Figure S1. Scatter plots of nitrate and ammonium in $PM_{2.5}$, PM_{10}, and TSP at PAY.

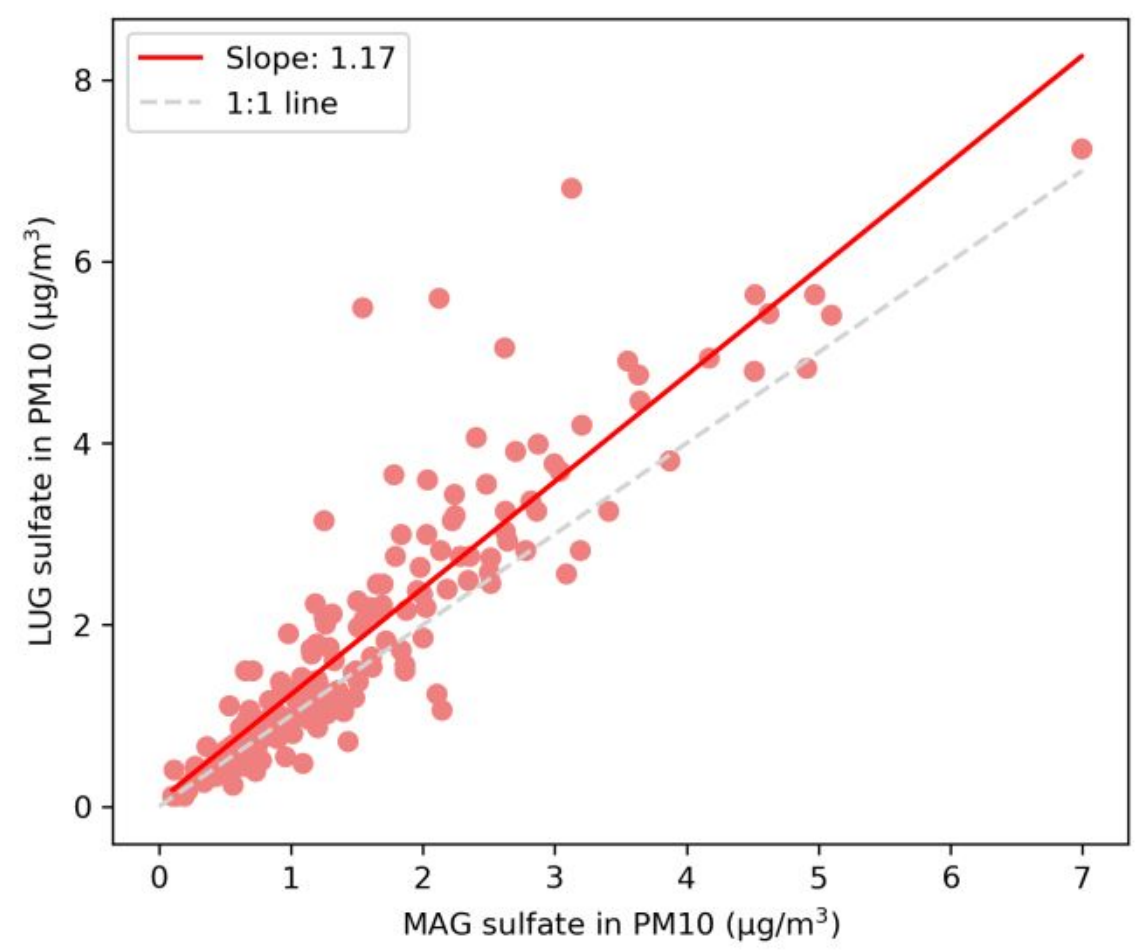

Figure S2. Scatter plot of sulfate concentrations at MAG and Lugano (LUG) for the periods August 2008 - July 2009 and June 2018- May 2019, based on measurements with a 4-day time resolution.

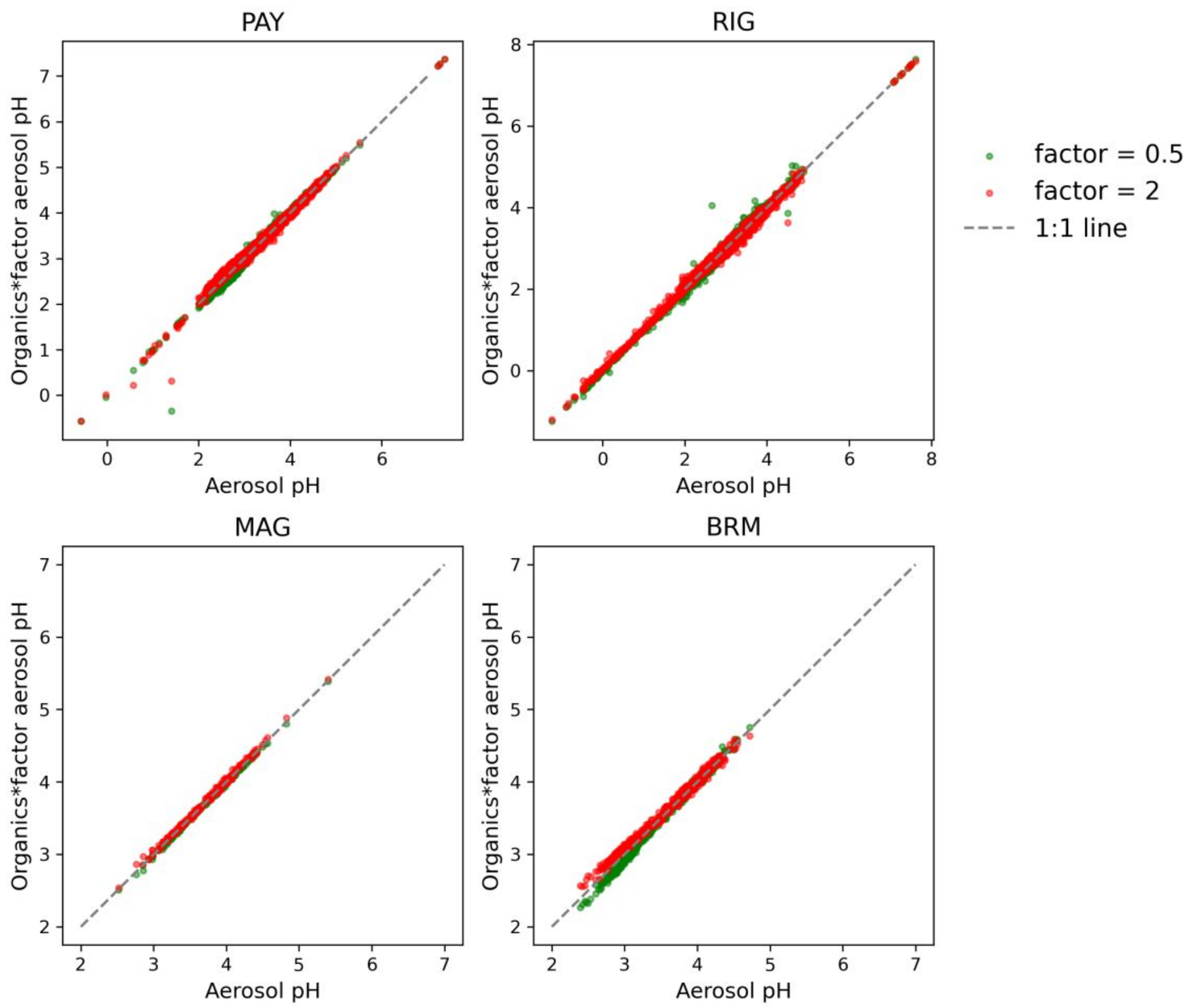

Figure S3. Sensitivity of aerosol pH to organic mass (OM) input. Aerosol pH was calculated using OM scaled by factors of 0.5 and 2, and compared to the baseline pH estimated using unadjusted OM values.

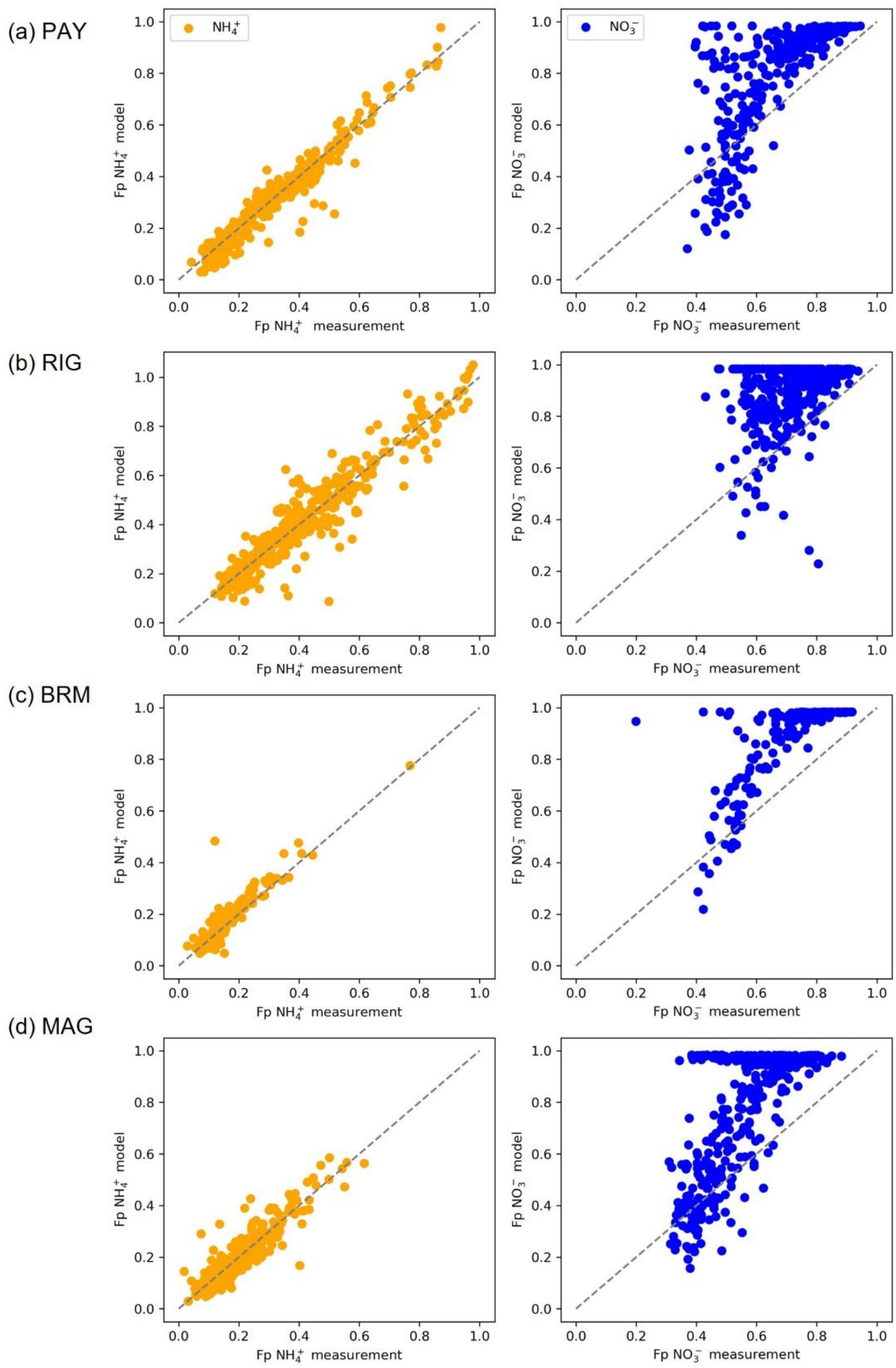

Figure S4. The agreements between measured and modelled particle phase fraction (Fp) of NH_4^+ and NO_3^- at four sampling sites. The grey dash lines are 1:1 line.

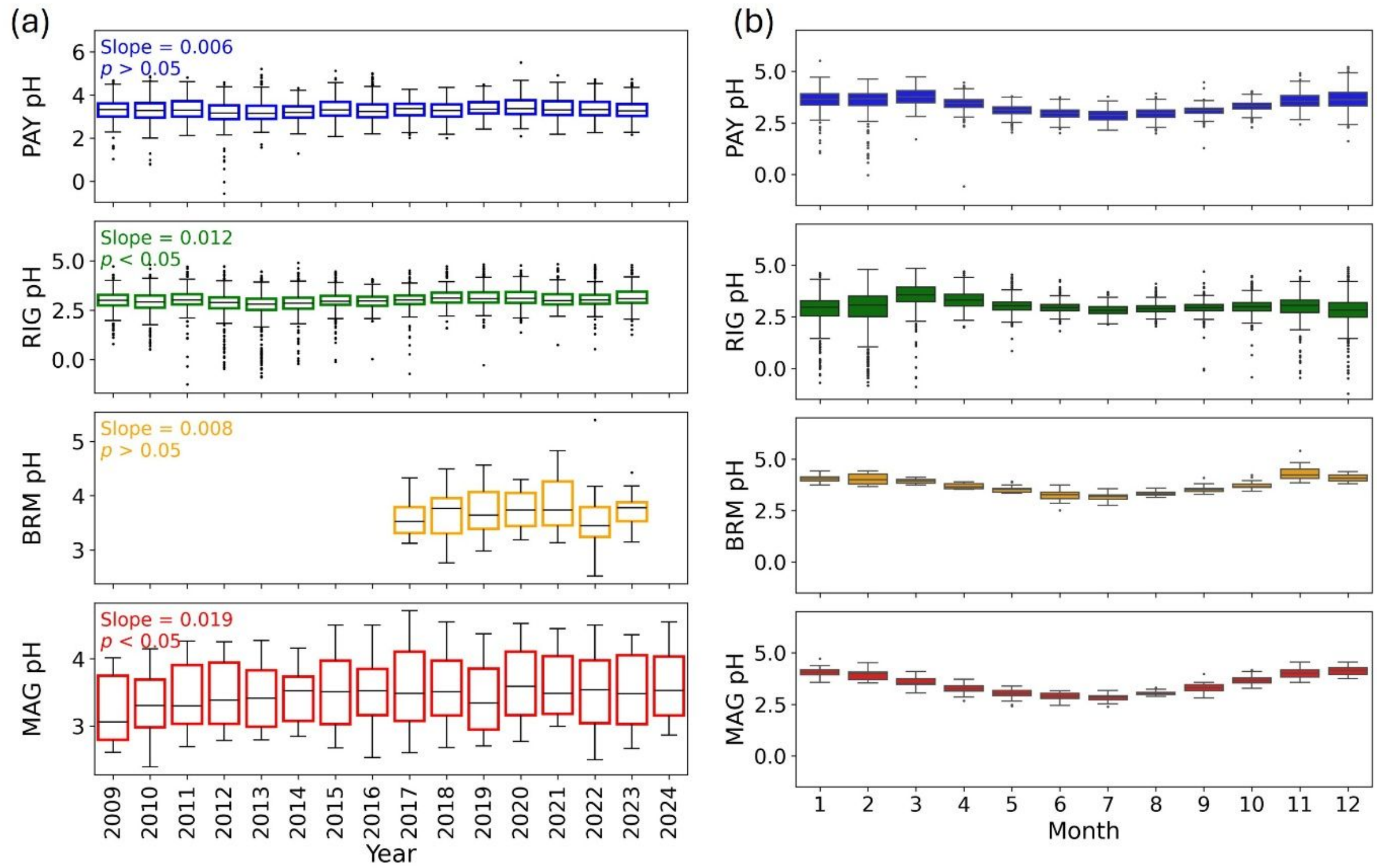

Figure S5. **(a) Yearly and (b) monthly variations of aerosol pH at PAY, RIG, BRM, and MAG.** The y axes are adjusted to show all data points.

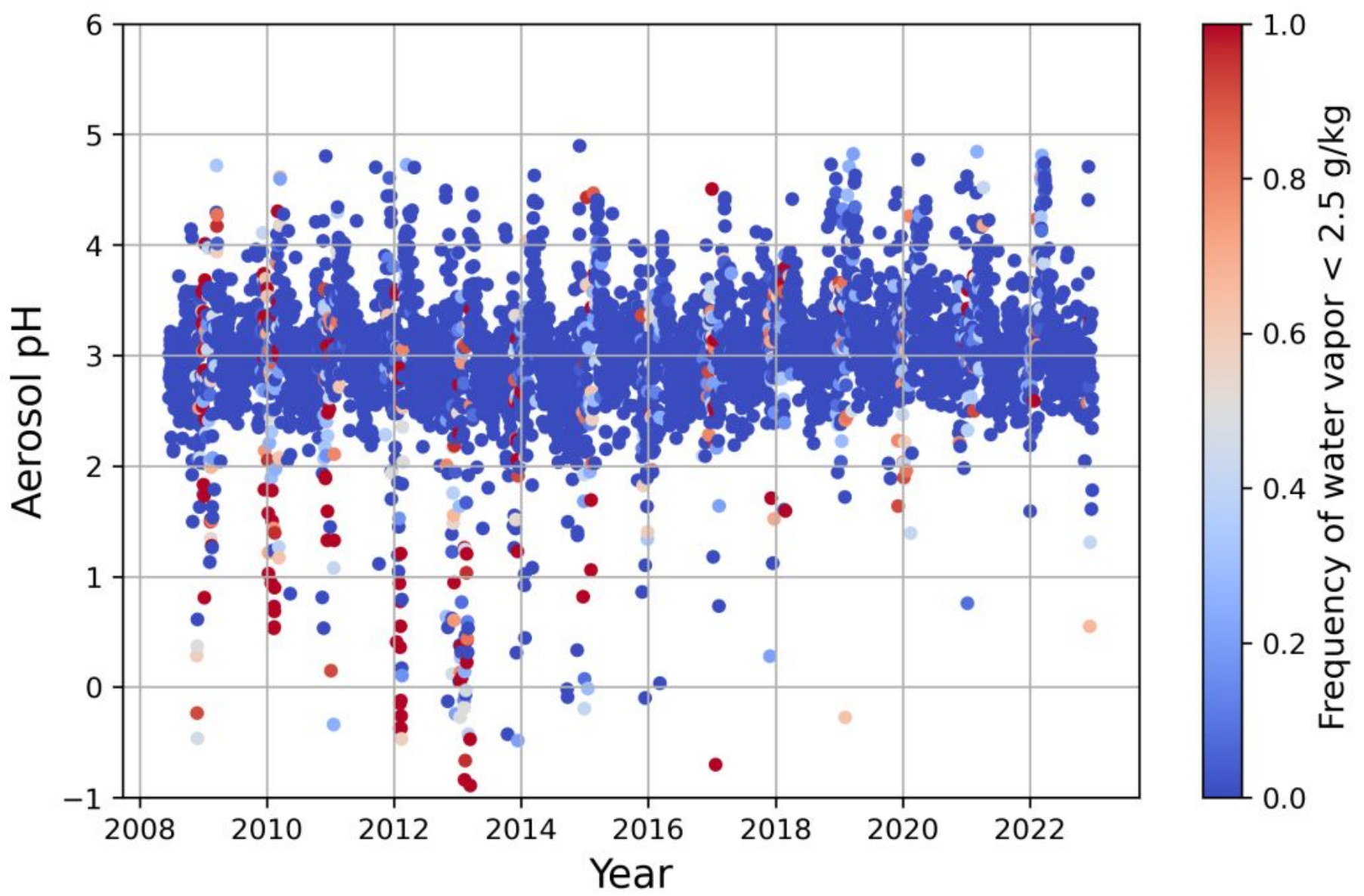

Figure S6. Aerosol pH at RIG over time at daily resolution. The aerosol pH is colored by the frequency of water vapor mixing ratio smaller than 2.5 g kg^{-1}, which indicate the likelihood of the influence of free-tropospheric air.

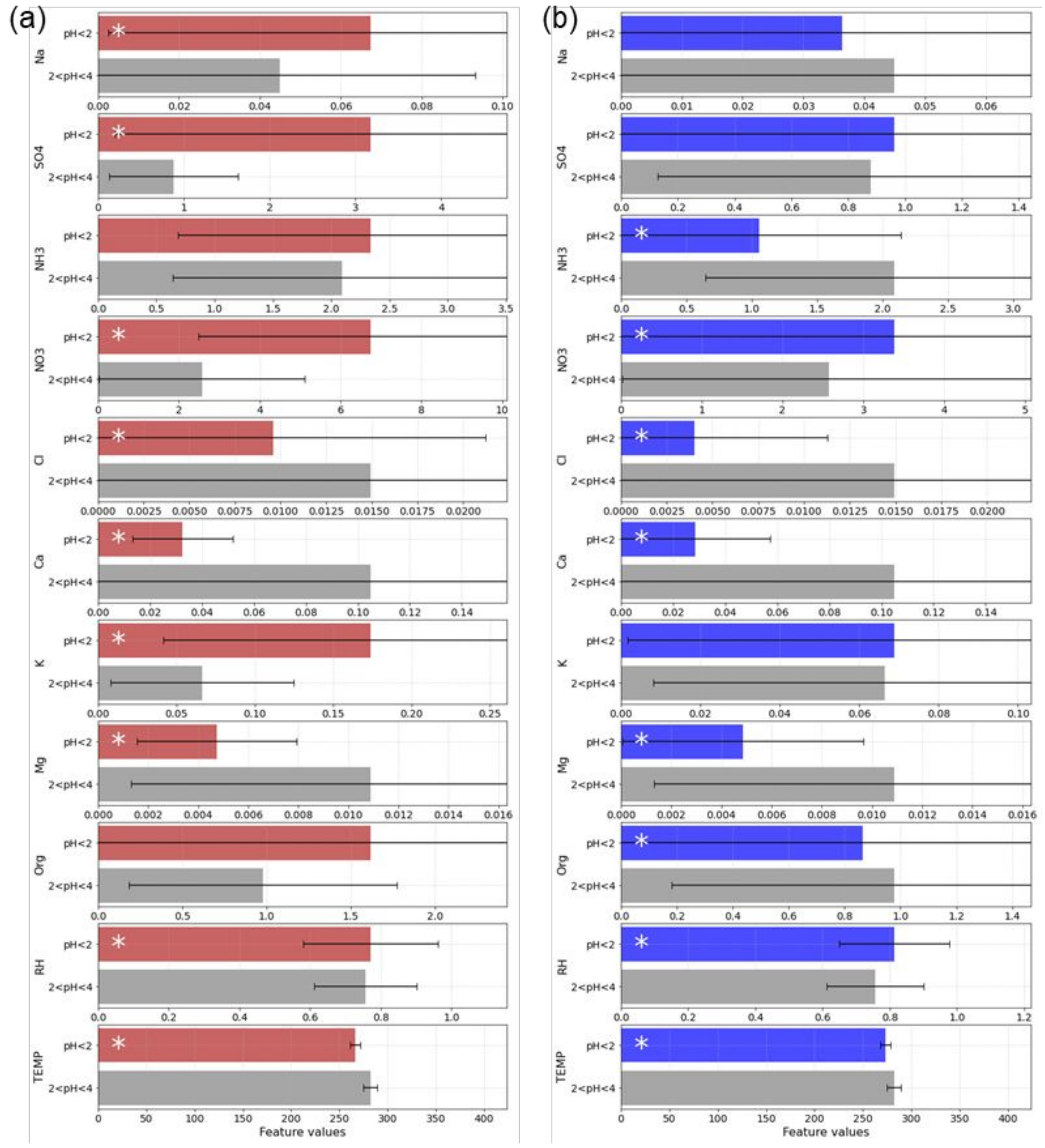

Figure S7. Comparison of input variables associated with aerosol pH < 2 and pH between 2 and 4 at RIG. (a) Under free tropospheric layer influence. (b) Within the planetary boundary layer. Variables marked with an asterisk (*) indicate statistically significant differences based on the Mann–Whitney U test ($p < 0.05$).

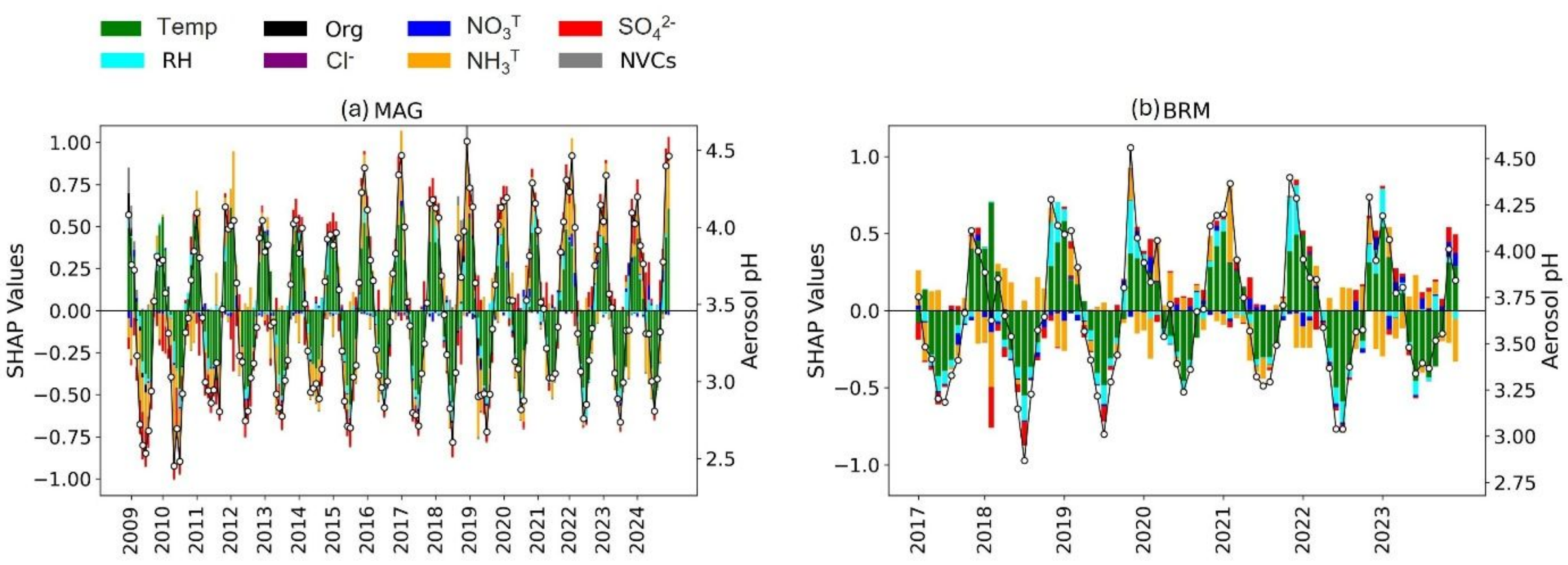

Figure S8. Monthly SHAP-derived contributions of key variables to aerosol pH at (a) MAG and (b) BRM, respectively**.** The SHAP values of temperature (Temp), relative humidity (RH), total nitrate

(NO_3^T), total ammonia (NH_3^T), sulfate (SO_4^{2-}), chloride (Cl^-), organics (Org), and non-volatile cations (NCVs) are shown as stacked bars.

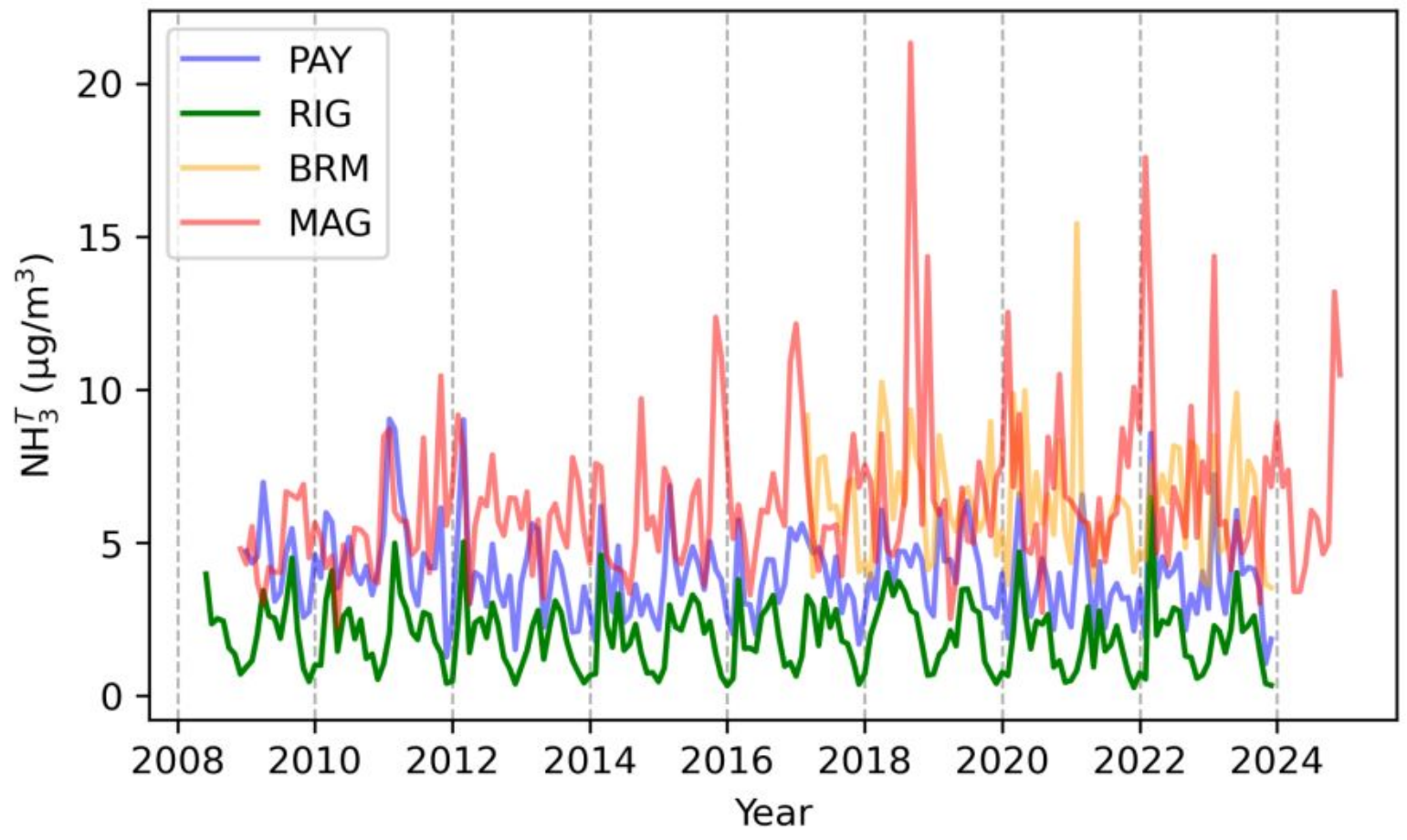

Figure S9. The time series of NH_3^T at four sampling sites.

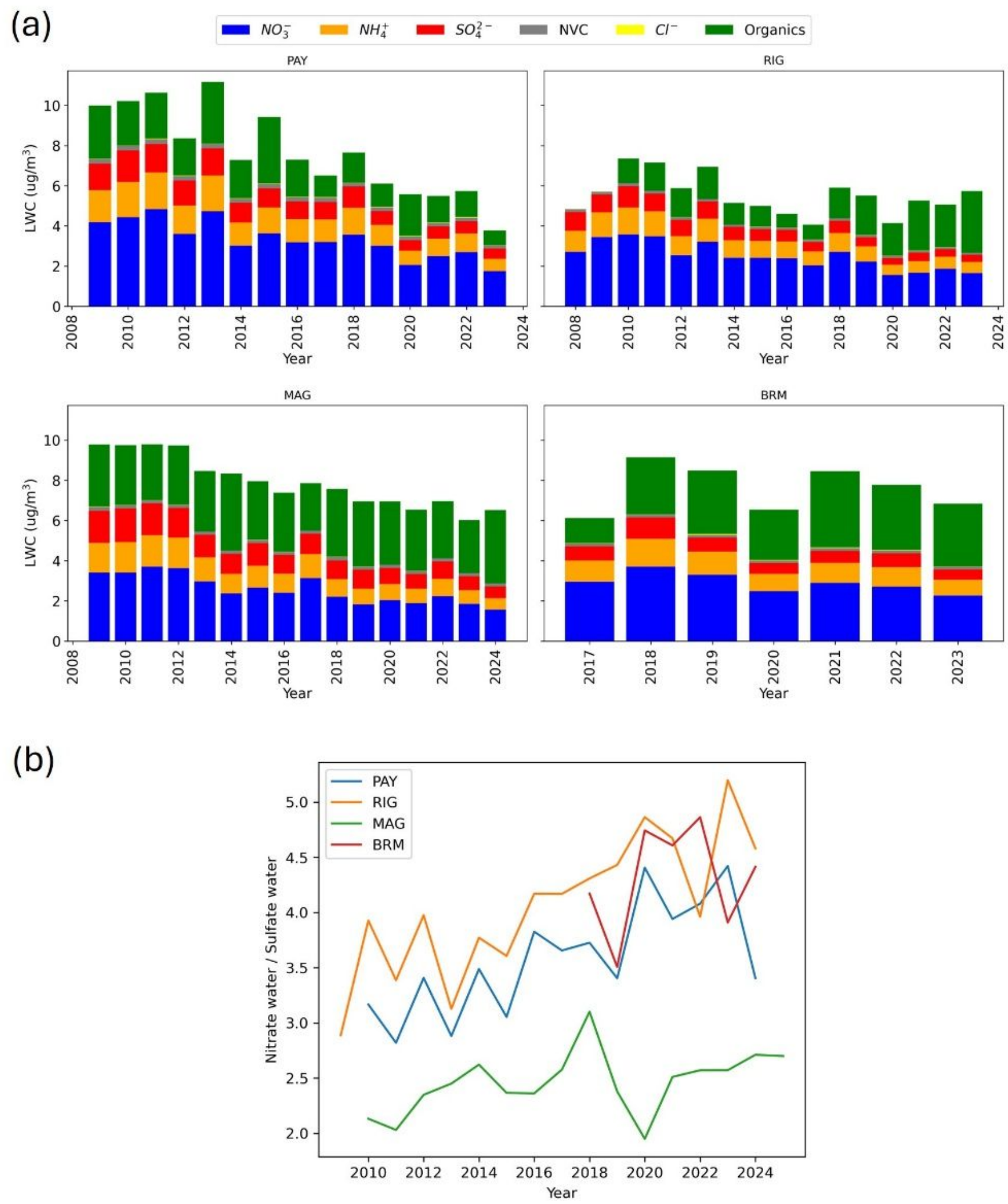

Figure S10. Panel (a): The yearly absolute contribution from aerosol chemical components to aerosol liquid water content (LWC). Panel (b): The ratio of water associated with nitrate and with sulfate at four sites.

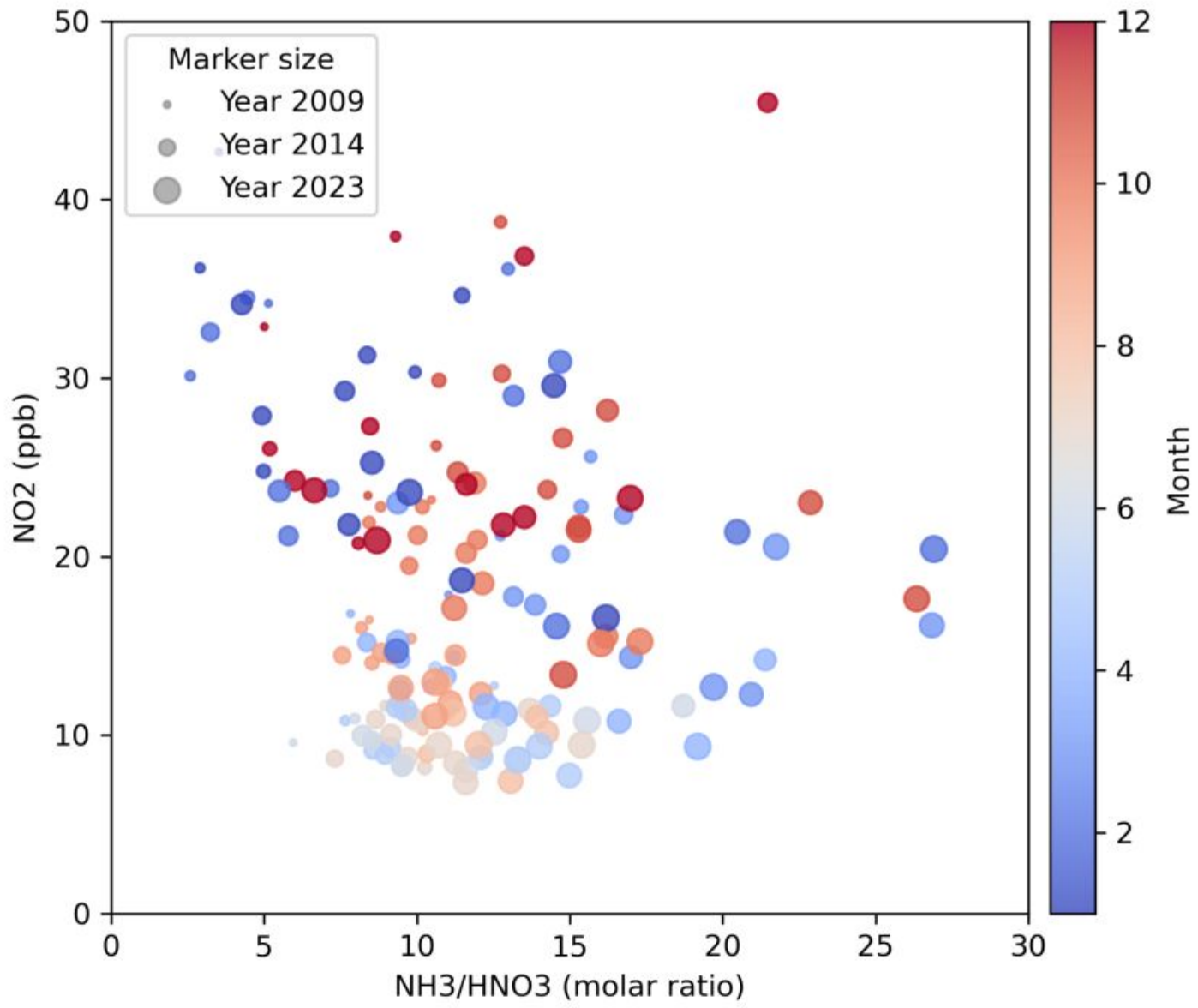

Figure S11. Scatter plot of the NH_3/HNO_3 molar ratio versus NO_2 at Payerne, based on monthly data from 2009 to 2023.
